# Comparison of the incidence of slow flow after rotational atherectomy with IVUS-crossable versus IVUS-uncrossable calcified lesions

**DOI:** 10.1038/s41598-020-68361-z

**Published:** 2020-07-09

**Authors:** Kenichi Sakakura, Yousuke Taniguchi, Kei Yamamoto, Takunori Tsukui, Masaru Seguchi, Hiroshi Wada, Shin-ichi Momomura, Hideo Fujita

**Affiliations:** Division of Cardiovascular Medicine, Saitama Medical Center, Jichi Medical University, 1-847 Amanuma, Omiya, Saitama, 330-8503 Japan

**Keywords:** Interventional cardiology, Cardiac device therapy

## Abstract

Although the usefulness of intravascular ultrasound (IVUS) in rotational atherectomy (RA) has been widely recognized, an IVUS catheter may not cross the target lesion because of severe calcification. The aim of this study was to compare the incidence of slow flow following RA between IVUS-crossable versus IVUS-uncrossable calcified lesions. We included 284 RA lesions, and divided into an IVUS-crossable group (n = 150) and an IVUS-uncrossable group (n = 134). The primary endpoint was slow flow just after RA. The incidence of slow flow (TIMI flow grade ≤ 2) was significantly greater in the IVUS-uncrossable group than in the IVUS-crossable group (26.1% vs. 10.7%, p = 0.001). The incidence of severe slow flow (TIMI grade ≤ 1) was also greater in the IVUS-uncrossable group than in the IVUS-crossable group (9.7% vs. 2.7%, p = 0.022). The multivariate logistic regression model showed a significant association between slow flow and pre-IVUS uncrossed lesions (vs. crossed lesions: odds ratio 2.103, 95% confidence interval 1.047–4.225, p = 0.037). In conclusion, the incidence of slow flow/severe slow flow just after RA was significantly greater in the IVUS-uncrossable lesions than in the IVUS-crossable lesions. Our study suggests the possibility that the IVUS-crossability can be used as a risk stratification of severe calcified lesions.

## Introduction

Rotational atherectomy (RA) is still a cornerstone for the treatment of coronary lesions with a high calcium content^1^. However, the incidence of severe complications is greater in percutaneous coronary interventions (PCI) with than without RA^2^, demanding further refinement in RA. Intravascular imaging devices including intravascular ultrasound (IVUS) and optical coherence tomography (OCT) can provide useful information regarding calcification such as depth, longitudinal length, and arch of calcification^3–6^. Therefore, intravascular imaging devices have been frequently used in current PCI with RA^7–9^. Moreover, the intravascular imaging may be helpful in the selection of initial burr sizes or RotaWires (BOSTON SCIENTIFIC, Marlborough, MA, USA), especially for operators with insufficient experiences in RA^10^.

Although the usefulness of intravascular imaging devices in RA has been widely recognized, an intravascular imaging device may not cross the target lesion because of severe calcification^11^. If an IVUS catheter cannot cross the lesion, operators have to decide the strategy of RA from angiographic findings by their own experiences, which may be a difficult situation for junior RA operators. Furthermore, if the incidence of complications following RA was greater in the IVUS-uncrossable lesions than in the IVUS-crossable lesions, junior RA operators would face high-risk lesions without imaging information, which could result in fatal complications. The aim of this study was to compare the incidence of slow flow following RA between IVUS-crossable versus IVUS-uncrossable calcified lesions.

## Methods

### Study design

**Figure 1:**
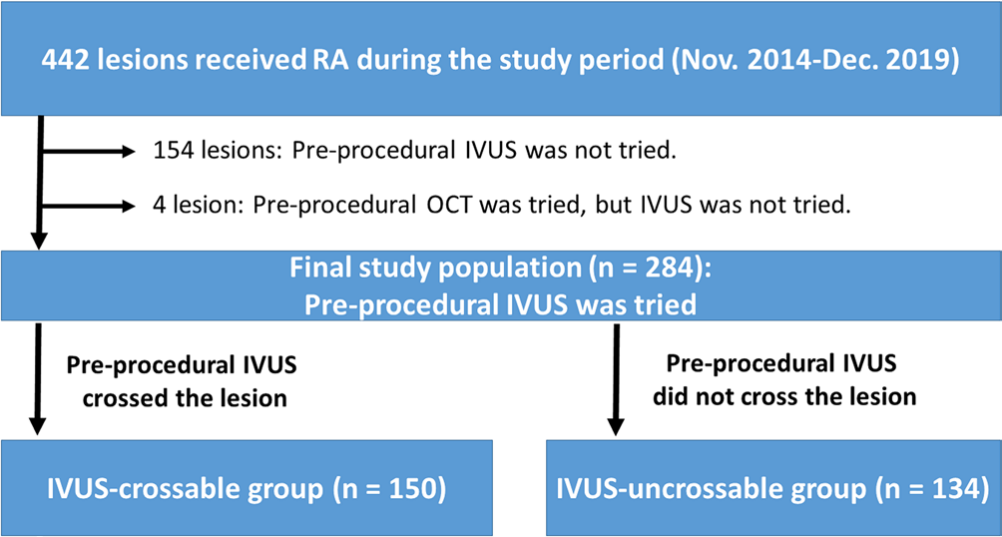
Study flow chart. *RA *rotational atherectomy, *IVUS *intravascular ultrasound, *OCT *optical coherence tomography.

This was a retrospective, single-center study. We reviewed 442 consecutive coronary lesions that were treated by RA in our institution during the period from November 2014 to December 2019. Indications for RA in our institution are the following: (1) angiographically moderate or severely calcified lesions, (2) diffuse lesions expected to be difficult to stent, and (3) ostial lesions^12,13^. We excluded 154 lesions in which pre-procedural IVUS was not attempted, and also excluded 4 lesions in which pre-procedural OCT was attempted but pre-procedural IVUS was not attempted. The final study consisted of 284 lesions, in which pre-procedural IVUS catheter was attempted before RA. The lesions were further classified into an IVUS-crossable group (n = 150) and an IVUS-uncrossable group (n = 134) according to the pre-procedural IVUS-crossability. The study flow chart is shown in Fig. 1. The primary endpoint was slow flow defined as transient thrombolysis in myocardial infarction (TIMI) flow grade ≤ 2 just after RA^14^. The secondary endpoint was severe slow flow defined as transient TIMI flow grade ≤ 1 just after RA. We also compared the incidence of ischemia-driven target vessel revascularization (TVR) between the 2 group. The study was approved by the institutional review board of the Saitama Medical Center, Jichi Medical University, and written informed consent was waved by the institutional review board of the Saitama Medical Center, Jichi Medical University, because of the retrospective study design. All methods were performed in accordance with the relevant guidelines and regulations.

### Acquisition of angiograms and evaluation of slow flow

The procedures were performed with a biplane fluoroscopy system [Artis zee BC (Model number 10094141, Model year 2009, March) and Artis zee BA (Model number 10094141, Model year 2013, January), SIEMENS, Munich, Germany] containing three magnetic fields and a standard image acquisition program at 15 frames per second during cine acquisition and 7.5 frames per second during fluoroscopy^15^. Thus, the evaluation of slow flow was done at 15 frames per second. Operators used a power injector (Zone master, Sugan Co., Ltd., Osaka, Japan) with predefined settings (total 7 ml, 3 ml/s for left coronary artery, total 5 ml, 2.5 ml/s for right coronary artery). However, operators modified the amount of contrast and injection speed to achieve sufficient images. The evaluation of TIMI-flow grade just after RA was performed by an unblinded operator (KS) like previous studies regarding RA from our institution^12–14,16^.

### Rotational atherectomy

RA was performed using standard techniques as previously described^11^. A nicorandil-based drug cocktail was used during RA to prevent slow flow^17^. We preferred to use ≥ 7-Fr guide catheters with side holes for RA. The lesion was crossed with a 0.014-inch conventional guidewire, and IVUS was attempted. The type of IVUS catheter was selected based on the discretion of the interventional cardiologist. Among 284 lesions, OptiCross (n = 222) (BOSTON SCIENTIFIC, Marlborough, MA, USA), Navifocus WR (n = 43) (TERUMO, Tokyo, Japan), AltaView (n = 15) (TERUMO, Tokyo, Japan), Eagle Eye (n = 3) (PHILLIPS VOLCANO, San Diego, CA, USA), and ViewIT (TERUMO, Tokyo, Japan) (n = 1) were used as the IVUS catheter. After the attempt of IVUS, a 0.014-inch conventional guidewire was exchanged with a 0.009-inch RotaWire floppy or RotaWire extra support guidewire (BOSTON SCIENTIFIC, Marlborough, MA, USA) using a microcatheter. The RA burr was subsequently advanced over the wire to a position proximal to the lesion. The initial rotational speed was set within the conventional range (140,000–190,000 rpm) with the burr proximal to the lesion, and several lesions were randomly allocated to 140,000 rpm or 190,000 rpm^13^. The burr was activated and moved forward with a slow pecking motion. Each run time was < 30 s, and care was taken to avoid a decrease in rotational speed > 5,000 rpm. However, the excessive speed down was sometimes observed especially in the ostium of right coronary artery^16^. The initial burr size was either 1.25-mm or 1.5-mm, which is supported by the European expert consensus on RA^18^. After the burr passed the lesion, the burr was removed using the dynaglide mode or trapping balloon technique^19^. The presence of coronary flow was confirmed by injecting sufficient contrast medium immediately after the burr was removed. Following RA, balloon dilatation was performed using a non-compliant balloon/scoring balloon/cutting balloon to facilitate stent implantation.

RA was not used as first-line therapy to treat culprit lesions in acute coronary syndrome (ACS); however, RA was used to treat culprit lesions in ACS if necessary^14^. Furthermore, an intra-aortic balloon pump (IABP) was inserted via a femoral artery before RA in high-risk cases such as those with severe left ventricular dysfunction, unprotected left main stenosis, or severe 3-vessel disease^11^. This was done because complications such as slow flow or peri-procedural myocardial infarction were more frequent in these high-risk cases^12^.

### Complications

We collected data on the following complications: slow flow just after RA, severe slow flow just after RA, vessel perforation (type III) due to the burr, burr entrapment, and peri-procedural myocardial infarction with slow flow. Peri-procedural myocardial infarction was defined as an increase in creatine kinase (at least three-fold above the normal upper limit)^12,13^.

### Definitions

Hypertension was defined as a systolic blood pressure > 140 mmHg, diastolic blood pressure > 90 mmHg, or medical treatment for hypertension^13^. Diabetes mellitus was defined as a hemoglobin A1c level > 6.5% or treatment for diabetes mellitus^13,20^. Hyperlipidemia was defined as a total cholesterol level > 220 mg/dl, a low-density lipoprotein cholesterol level > 140 mg/dl, or treatment for hyperlipidemia^13^. eGFR was calculated using the MDRD formula^21^. ACS was defined as ST-segment elevation myocardial infarction, non-ST-segment elevation myocardial infarction, or unstable angina^13^. The reference diameter and lesion length were calculated by quantitative coronary angiography. Offline, computer-based software QAngio XA 7.3 (MEDIS Imaging Systems, Leiden, The Netherlands) was used for quantitative coronary angiography^11^. The burr-to-artery ratio was defined as the burr size divided by the reference diameter^11^.

### Statistical analysis

Data are presented as a percentage for categorical variables and the mean ± SD for continuous variables. The Wilk–Shapiro test was performed to determine if the continuous variables were normally distributed. Normally distributed continuous variables were compared between the 2 groups using a Student’s *t* test. Otherwise, continuous variables were compared using a Mann–Whitney *U* test. Categorical data were compared using a Fischer’s exact test. We performed multivariate stepwise logistic regression analysis to investigate the association between IVUS-crossability and slow flow. In the model 1, the dependent variable was slow flow just after RA. Variables that had a significant association (P < 0.05) between the 2 groups were used as independent variables. The multivariate logistic regression analysis with Wald Statistical criteria using backward elimination method was performed. In the model 2, the dependent variable was severe slow flow just after RA. Variables that had a significant association (P < 0.05) between the 2 groups were used as independent variables. The multivariate logistic regression analysis with Wald Statistical criteria using backward elimination method was performed. Odds ratios (OR) and the 95% confidence intervals (CI) were calculated. Ischemia-driven TVR-free survival curves were constructed using the Kaplan–Meier method, and the statistical difference between curves was assessed by the log-lank test. All reported P-values were determined by two-sided analysis, and P-values < 0.05 were considered significant. All analyses were performed with IBM SPSS statistics version 25 (Chicago, IL, USA).

## Results

The comparison of patients and lesion characteristics between the 2 groups are summarized in Table 1. The prevalence of male sex was significantly less in the IVUS-uncrossable group than in the IVUS-crossable group (65.7% vs. 76.7%, p = 0.048). The prevalence of diabetes mellitus was significantly greater in the IVUS-uncrossable group than in the IVUS-crossable group (65.7% vs. 52.3%, p = 0.029). The culprit lesion in acute coronary syndrome was more frequently observed in the IVUS-uncrossable group than in the IVUS-crossable group (26.1% vs. 14.0%, p = 0.011). Left main-left anterior descending artery lesions were less frequently observed in the IVUS-uncrossable group than in the IVUS-crossable group (p = 0.015). The reference diameter was significantly smaller in the IVUS-uncrossable group than in the IVUS-crossable group (2.26 ± 0.59 mm vs. 2.54 ± 0.61 mm, p < 0.001). The lesion length was significantly longer in the IVUS-uncrossable group than in the IVUS-crossable group (27.40 ± 15.65 mm vs. 22.99 ± 15.47 mm, p = 0.008). Moderate to severe angulation was more frequently observed in the IVUS-uncrossable group than in the IVUS-crossable group (p = 0.002).

**Table 1:** Comparison of patients and lesions characteristics between the IVUS-crossable group and IVU-uncrossable group.

	All (n = 284)	IVUS-crossable group (n = 150)	IVUS-uncrossable group (n = 134)	P value
**Patient characteristics**				
Age (years)	73.9 ± 8.8	74.7 ± 8.0	73.0 ± 9.5	0.176
Men—n, (%)	203 (71.5)	115 (76.7)	88 (65.7)	0.048
Overweight (BMI ≥ 25 kg/m^2^)—n, (%)	79 (27.8)	38 (25.3)	41 (30.6)	0.354
Hypertension—n, (%)	274 (96.5)	143 (95.3)	131 (97.8)	0.343
Diabetes mellitus—n, (%) (n = 283)	166 (58.7)	78 (52.3)	88 (65.7)	0.029
Hyperlipidemia—n, (%)	265 (93.3)	139 (92.7)	126 (94.0)	0.813
Current smoker—n, (%) (n = 282)	44 (15.6)	24 (16.0)	20 (15.2)	0.871
Chronic renal failure (creatinine > 2 mg/dl)—n, (%)	73 (25.7)	33 (22.0)	40 (29.9)	0.137
Estimated GFR (ml/min/1.73 m^2^)	65.0 ± 40.6	67.0 ± 38.7	62.8 ± 42.6	0.431
Chronic renal failure on hemodialysis—n, (%)	65 (22.9)	32 (21.3)	33 (24.6)	0.572
Statin treatment—n, (%)	262 (92.3)	138 (92.0)	124 (92.5)	1.000
**Lesion characteristics**				
Culprit lesion in acute coronary syndrome—n, (%)	56 (19.7)	21 (14.0)	35 (26.1)	0.011
In-stent lesion—n, (%)	19 (6.7)	14 (9.3)	5 (3.7)	0.094
Target coronary artery				0.015
Left main- left anterior descending artery—n, (%)	190 (66.9)	111 (74.0)	79 (59.0)	
Left circumflex artery—n, (%)	17 (6.0)	5 (3.3)	12 (9.0)	
Right coronary artery—n, (%)	77 (27.1)	34 (22.7)	43 (32.1)	
**Specific target coronary artery**				
Ostial left main—n, (%)	2 (0.7)	2 (1.3)	0	0.500
Ostial left anterior descending artery—n, (%)	36 (12.7)	24 (16.0)	12 (9.0)	0.107
Ostial left circumflex artery—n, (%)	4 (1.4)	3 (2.0)	1 (0.7)	0.625
Ostial right coronary artery—n, (%)	23 (8.1)	12 (8.0)	11 (8.2)	1.000
Reference diameter (mm)	2.41 ± 0.61	2.54 ± 0.61	2.26 ± 0.59	< 0.001
Lesion length (mm)	25.07 ± 15.68	22.99 ± 15.47	27.40 ± 15.65	0.008
Initial TIMI flow grade 3—n, (%)	252 (88.7)	137 (91.3)	115 (85.8)	0.188
Lesion angle				0.002
Mild angulation (< 30°)	143 (50.4)	88 (58.7)	55 (41.0)	
Moderate angulation (30°–60°)	112 (39.4)	54 (36.0)	58 (43.3)	
Severe angulation (≥ 60°)	29 (10.2)	8 (5.3)	21 (15.7)	
Angiographically severe calcification	278 (97.9)	147 (98.0)	131 (97.8)	1.000

Data are expressed as the mean ± SD or number (percentage). A Mann–Whitney *U* test was used for continuous variables, and a Fischer’s exact test was used for categorical variables.

*GFR *glomerular filtration rate.

The comparison of procedural characteristics between the 2 groups is summarized in Table 2. The guidewire switch was more frequently performed in the IVUS-uncrossable group than in the IVUS-crossable group (p = 0.003). The 1.25-mm burr was more frequently used as an initial burr in the IVUS-uncrossable group than the IVUS-crossable group (39.6% vs. 15.3%, p < 0.001). The final burr size was smaller in the IVUS-uncrossable group than in the IVUS-crossable group (p < 0.001). Initial burr-to-artery ratio was significantly greater in the IVUS-uncrossable group than in the IVUS-crossable group (0.66 ± 0.16 vs. 0.61 ± 0.15, p = 0.008). Total run time, single run time, rotational speed, and maximum speed reduction during RA were significantly greater in the IVUS-uncrossable group than in the IVUS-crossable group.

**Table 2:** Comparison of procedural characteristics between the IVUS-crossable group and IVU-uncrossable group.

	All (n = 284)	IVUS-crossable group (n = 150)	IVUS-uncrossable group (n = 134)	P value
**Procedural characteristics**				
Guiding catheter size and system				0.081
6Fr—n, (%)	4 (1.4)	0 (0)	4 (3.0)	
7Fr—n, (%)	257 (90.5)	136 (90.7)	121 (90.3)	
8Fr—n, (%)	23 (8.1)	14 (9.3)	9 (6.7)	
Intra-aortic balloon pump support—n, (%)	20 (7.0)	11 (7.3)	9 (6.7)	1.000
Any balloon dilatation or try to balloon dilatation before RA—n, (%)	28 (9.9)	11 (7.3)	17 (12.7)	0.163
Guidewire used during rotational atherectomy				0.003
RotaWire floppy—n, (%)	216 (76.1)	122 (81.3)	94 (70.1)	
RotaWire extra support—n, (%)	40 (14.1)	22 (14.7)	18 (13.4)	
Guidewire switch from floppy to extra support—n, (%)	23 (8.1)	6 (4.0)	17 (12.7)	
Guidewire switch from extra support to floppy—n, (%)	5 (1.8)	0 (0)	5 (3.7)	
Number of burrs used	1.2 ± 0.5	1.3 ± 0.5	1.2 ± 0.5	0.554
Initial burr size				< 0.001
1.25-mm	76 (26.8)	23 (15.3)	53 (39.6)	
1.5-mm	208 (73.2)	127 (84.7)	81 (60.4)	
Final burr size				< 0.001
1.25-mm	66 (23.2)	17 (6.0)	49 (17.3)	
1.5-mm	173 (60.9)	100 (66.7)	73 (54.5)	
1.75-mm	12 (4.2)	8 (5.3)	4 (3.0)	
2.0-mm	33 (11.6)	25 (16.7)	8 (6.0)	
Initial burr-to-artery ratio	0.63 ± 0.16	0.61 ± 0.15	0.66 ± 0.16	0.008
Final burr-to-artery ratio	0.66 + 0.17	0.65 + 0.16	0.68 + 0.18	0.163
Total run time (s)	91.8 ± 73.2	72.8 ± 59.5	115.1 ± 80.6	< 0.001
Mean single run time (s)	12.8 ± 3.1	11.9 ± 2.6	13.7 ± 3.4	< 0.001
Mean rotational speed (× 1,000 rpm)	173.2 ± 10.1	171.6 ± 10.6	175.0 ± 9.1	0.021
Maximum speed reduction during rotational atherectomy (rpm) (n = 281)	6,630 ± 5,166	6,228 ± 6,007	7,083 ± 3,987	0.001
Systolic blood pressure just before rotational atherectomy (mmHg)	153 + 27	153 + 28	153 + 26	0.919
Diastolic blood pressure just before rotational atherectomy (mmHg)	75 + 14	76 + 15	75 + 13	0.584
Heart rate just before rotational atherectomy (per minute)	71 + 14	71 + 12	72 + 15	0.657
Final procedure				0.441
Rotational atherectomy + balloon including drug-coating balloon—n, (%)	23 (8.1)	15 (10.0)	8 (6.0)	
Rotational atherectomy + bare-metal stent—n, (%)	3 (1.1)	2 (1.3)	1 (0.7)	
Rotational atherectomy + drug-eluting stent—n, (%)	257 (90.5)	133 (88.7)	124 (92.5)	
Rotational atherectomy + covered stent for perforation—n, (%)	1 (0.4)	0 (0)	1 (0.7)	

Data are expressed as the mean ± SD or number (percentage). A Mann–Whitney *U* test was used for continuous variables, and a Fischer’s exact test was used for categorical variables.

*GFR *glomerular filtration rate.

The comparison of complications between the 2 groups is shown in Table 3. The incidence of slow flow just after RA was significantly greater in the IVUS-uncrossable group than in the IVUS-crossable group (26.1% vs. 10.7%, p = 0.001). The incidence of severe slow flow just after RA was also greater in the IVUS-uncrossable group than in the IVUS-crossable group (9.7% vs. 2.7%, p = 0.022). The incidence of periprocedural MI was not different between the 2 groups.

**Table 3:** Comparison of complications between the IVUS-crossable group and IVU-uncrossable group.

	All (n = 284)	IVUS-crossable group (n = 150)	IVUS-uncrossable group (n = 134)	P value
Slow flow (≤ TIMI-2) just after RA	51 (18.0)	16 (10.7)	35 (26.1)	0.001
Severe slow flow (≤ TIMI-1) just after RA	17 (6.0)	4 (2.7)	13 (9.7)	0.022
Periprocedural MI with slow flow	4 (1.4)	1 (0.7)	3 (2.2)	0.346
Final TIMI flow grade ≤ 2	2 (0.7)	0	2 (1.5)	0.222
Vessel perforation (Type III) due to Burr	1 (0.4)	0 (0)	1 (0.7)	0.472
Burr entrapment	1 (0.4)	1 (0.7)	0 (0)	1.000

Data are expressed as the number (percentage). A Fischer’s exact test was used to compare the 2 groups.

*TIMI *thrombolysis in myocardial infarction.

**Figure 2:**
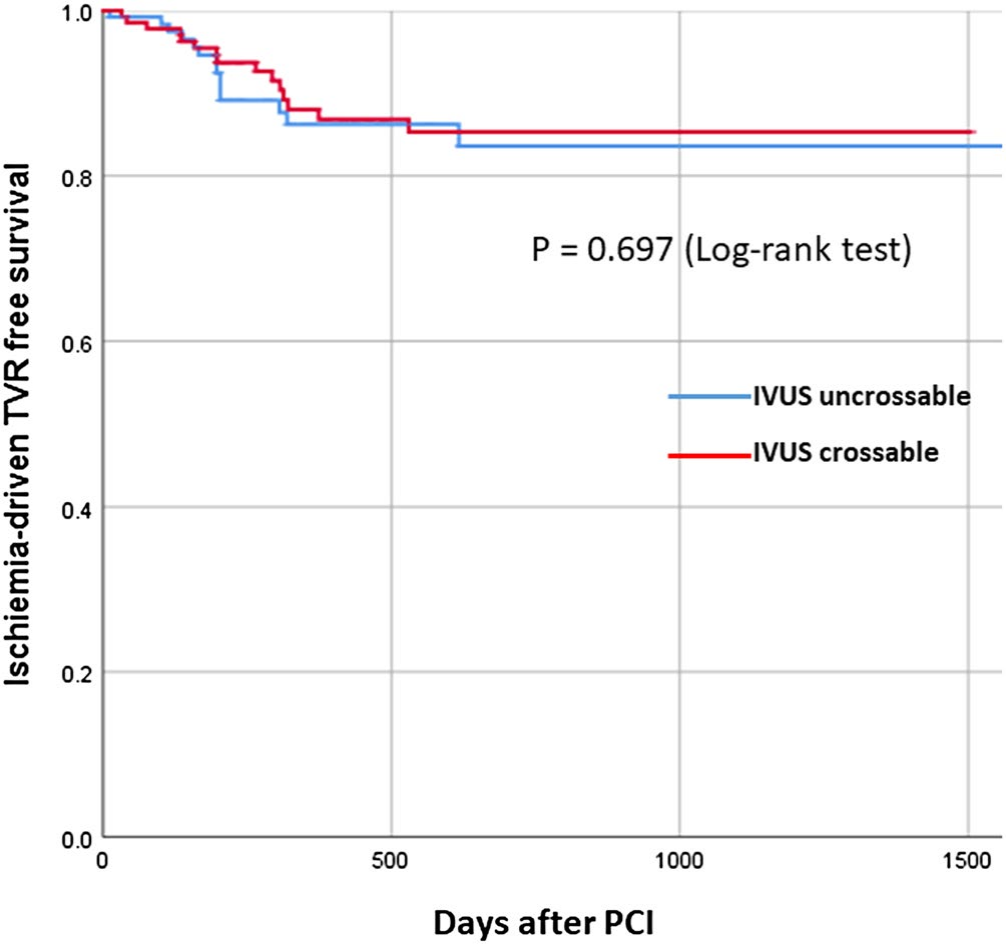
Kaplan–Meier curves of ischemia-driven TVR free survival.

The multivariate logistic regression models to investigate the association between IVUS crossability and slow flow/severe slow flow are shown in Table 4. In the model 1, the initial model included male sex, diabetes mellitus, culprit lesion in acute coronary syndrome, target lesion (left main- left anterior descending artery vs. others), lesion length, severe angulation, RotaWire floppy as an initial wire, initial burr-to-artery ratio, and pre-IVUS uncrossed lesions (vs. crossed lesions) as independent variables. The final model showed a significant association between slow flow and pre-IVUS uncrossed lesions (vs. crossed lesions: OR 2.103, 95% CI 1.047–4.225, p = 0.037). In the model 2, the initial model also included male sex, diabetes mellitus, culprit lesion in acute coronary syndrome, target lesion (left main- left anterior descending artery vs. others), lesion length, severe angulation, RotaWire floppy as an initial wire, initial burr-to-artery ratio, and pre-IVUS uncrossed lesions (vs. crossed lesions) as independent variables. The final model showed a significant association between severe slow flow and pre-IVUS uncrossed lesions (vs. crossed lesions: OR 3.312, 95% CI 1.036–10.589, p = 0.043). Figure 2 shows the Kaplan–Meier curves of ischemia-driven TVR-free survival. The median follow-up duration was 298 days (Q1-Q3: 177–620 days). Ischemia-driven TVR free survival curves were not different between the 2 groups (p = 0.697).

**Table 4:** Multivariate stepwise logistic regression model to investigate the association between pre-IVUS crossability and slow flow.

Dependent variable: Slow flow			
Independent variables	Odds ratio	95% confidence interval	P value
**Model 1: Dependent variable: Slow flow (≤ TIMI-2) just after RA**			
Lesion length (every 5 mm increase)	1.098	0.987–1.221	0.086
Severe angulation (≥ 60°)	2.767	1.088–7.041	0.033
Initial burr-to-artery ratio (every 0.1 increase)	1.581	1.271–1.966	< 0.001
Pre-IVUS uncrossed lesions (vs. pre-IVUS crossed lesions)	2.103	1.047–4.225	0.037

Dependent variable: Severe slow flow			
Independent variables	Odds ratio	95% confidence interval	P value
**Model 2: Dependent variable: Severe slow flow (≤ TIMI-1) just after RA**			
Initial burr-to-artery ratio (every 0.1 increase)	1.392	1.057–1.833	0.018
Pre-IVUS uncrossed lesions (vs. pre-IVUS crossed lesions)	3.312	1.036–10.589	0.043

The initial model included male sex, diabetes mellitus, culprit lesion in acute coronary syndrome, target lesion (left main- left anterior descending artery vs. others), lesion length, severe angulation, RotaWire floppy as an initial wire, initial burr-to-artery ratio, pre-IVUS uncrossed lesions (vs. crossed lesions). The multivariate logistic regression analysis with Wald Statistical criteria using backward elimination method was performed.

## Discussion

A total of 284 severely calcified lesions for which pre-procedural IVUS was attempted before RA were included in the present study, and were divided into an IVUS-crossable group (n = 150) and a IVUS-uncrossable group (n = 134), according to the IVUS-crossability. The incidence of slow flow/severe slow flow just after RA was significantly greater in the IVUS-uncrossable group than in the IVUS-crossable group. The multivariate logistic regression analysis confirmed the significant association between slow flow/severe slow flow and IVUS-crossability. Our results suggest that special attention should be paid to the IVUS-uncrossable lesions to prevent complications following RA.

Recently, many groups rigorously published research articles regarding RA with intravascular imaging devices^3,4,6–9^. Moreover, several review articles explain the interpretation of intravascular imaging findings in RA to facilitate use of imaging devices in RA^1,5,10^. However, there are few literatures focusing on the situation when an intravascular imaging device could not cross the severely calcified lesion before RA. As we shown in the present study, a substantial number of severely calcified lesions did not allow intravascular imaging devices to cross the lesion before RA. Furthermore, if we utilize IVUS correctly, the incidence of severe complications has been considered to lower in RA with than without IVUS^10^. However, the US National Inpatient Sample data set for the years 2012 to 2014 showed the higher incidence of iatrogenic and cardiac complications in IVUS-assisted atherectomy^22^, which would include IVUS-uncrossable lesions. It should be important to discuss strategies for severely calcified lesions that intravascular image devices cannot cross, especially for operators with insufficient RA experiences.

The reason why the incidence of slow flow was greater in the IVUS-uncrossable lesions than in the IVUS-crossable lesions should be discussed. The IVUS-uncrossable group had more complex features such as smaller reference diameter, longer lesion length, and severer angulation. Although the initial burr size was smaller in the IVUS-uncrossable group, initial burr-to-artery ratio was bigger in the IVUS-uncrossable group. Because the longer lesion length or bigger burr-to-artery ratio was known to be associated with the incidence of slow flow^23,24^, the IVUS-uncrossable group had greater risk of slow flow before RA as compared to the IVUS-crossable group. Moreover, the IVUS-uncrossable group had greater total run time, single run time, rotational speed, and maximum speed reduction during RA as compared to the IVUS-crossable group, which also supports that the lesion complexity was greater in the IVUS-uncrossable group than in the IVUS-crossable group.

The clinical implications of the present study should be noted. First, our study suggests the possibility that the IVUS-crossability can be used as a risk stratification of severe calcified lesions. If the pre-procedural IVUS catheters could not cross the lesion, operators should prepare the occurrence of slow flow after RA. In other words, operators may prepare intracoronary vasodilator such as nitroprusside, intravenous vasopressor such as noradrenaline, or intra-aortic balloon pumping if patient’s cardiac function was severely reduced. Moreover, such greater risk should be shared with other staffs such as nurses or medical engineers in the catheter laboratory to perform timely management for slow flow. Second, our results may be useful for establishing an educational program for junior RA operators. Because many literatures regarding RA and imaging devices have been published^1,3–10^, even junior RA operators may be able to select an appropriate burr size, RotaWire, and RA strategy as long as an imaging device can cross the lesion before RA. However, as we shown here, there were many lesions that an IVUS catheter cannot cross, and the incidence of slow flow was greater in IVUS-uncrossable lesions. Immediate RA to IVUS-uncrossable lesions may be appropriate for senior RA operators, but may not for junior RA operators. We should discuss whether junior RA operators should try immediate RA to such tough lesions or try small balloon dilatation followed by RA to avoid complications. Moreover, as we showed in the multivariate logistic regression analysis, the diffuse long lesion, severe angulation, and initial burr-to-artery ratio were associated with slow flow. Senior RA operators may anticipate the risk of complications from those angiographic findings without IVUS findings, and select small burrs for lesions with small reference diameter to keep appropriate burr-to-artery ratio. However, since those parameters (length, angle, diameter, or ratio) are continuous variables, it would be difficult for junior RA operators to anticipate the risk of complications without established cut-off values. For example, the recommended burr-to-artery ratio varies widely between European Consensus document (0.6) and North American Expert Review (0.4–0.6)^1,18^. Since the IVUS-crossability is a simple categorical variable (yes/no), it would be easy for junior RA operators to understand the risk of complications. Furthermore, if there were several RA operators in a catheter laboratory, a senior RA operator would actively assist a junior RA operator to perform RA to the IVUS-uncrossable lesion. Those discussions should be incorporated into the educational program for junior RA operators for better patient’s outcomes.

### Study limitations

Because our study was designed as a single-center, retrospective, observational study, there is a risk of patient selection bias and group-selection bias. Although vessel perforation and burr entrapment are unique complications in RA, our study population was too small to evaluate the difference in those complications between the 2 groups. Since we potentially recognized the greater risk of slow flow in IVUS-uncrossable lesions, we might be more careful to perform RA to IVUS-uncrossable lesions. In fact, we used smaller initial burrs for IVUS-uncrossable lesions. Nevertheless, the incidence of slow flow was greater in IVUS-uncrossable lesions, which supports the strong relationship between slow flow and IVUS-uncrossable lesions. The study endpoint (slow flow) might be influenced by various factors such as settings of power injectors, presence of side holes in guide catheters, and an unblinded evaluator (KS), which would limit reproducibility of the present study. Furthermore, we excluded 158 lesions in which pre-procedural IVUS was not attempted. Although we tended to skip pre-procedural IVUS in our early study period (until 2016), we routinely performed pre-procedural IVUS in our late study period, partly because a dedicated trapping balloon device (Kusabi: KANEKA, Osaka, Japan) facilitated the exchange of guidewires using microcatheters. In the multivariate logistic regression analysis, although we tried to avoid co-linearity of the independent variables, we could not confirm co-linearity of the variables statistically, because the main variable (IVUS-crossability) was a categorical variable (yes/no). Finally, our catheter laboratory rarely used OCT before RA. In fact, of 442 RA lesions during the study period, only 4 lesions (0.9%) received pre-procedural OCT. We decided to exclude those OCT cases from the final study population, because of our limited experiences with OCT.

## Conclusion

The incidence of slow flow/severe slow flow just after RA was significantly greater in the IVUS-uncrossable lesions than in the IVUS-crossable lesions. Our study suggests the possibility that the IVUS-crossability can be used as a risk stratification of severe calcified lesions in RA.

## Data Availability

All data are available from the corresponding author on reasonable request.
